# Supplementary material for: Harnessing fusion of genome-edited human stem cells to rapidly screen for novel protein functions in vivo
Source: bioRxiv. 2025 Jun 27:2025.06.25.661608. Preprint. [Version 1] doi: 10.1101/2025.06.25.661608 (PMC12262239; doi:10.1101/2025.06.25.661608)
Supplement: 1 [file NIHPP2025.06.25.661608V1-supplement-1.pdf]

bioRxiv preprint doi: https://doi.org/10.1101/2025.06.25.661608; this version posted June 27, 2025. The copyright holder for this preprint (which was not certified by peer review) is the author/funder, who has granted bioRxiv a license to display the preprint in perpetuity. It is made available under aCC-BY 4.0 International license.

Supplemental Figures

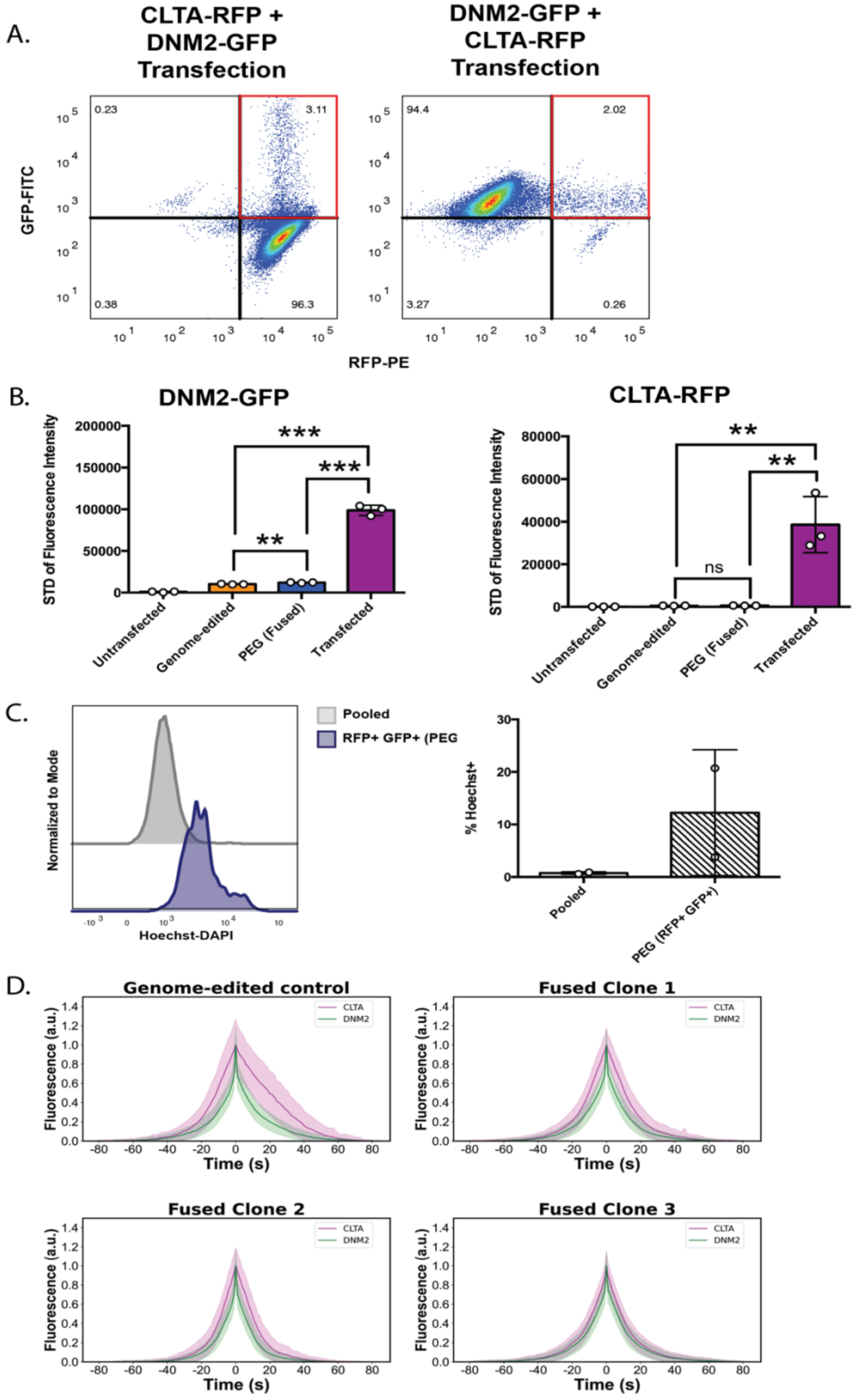

bioRxiv preprint doi: https://doi.org/10.1101/2025.06.25.661608; this version posted June 27, 2025. The copyright holder for this preprint (which was not certified by peer review) is the author/funder, who has granted bioRxiv a license to display the preprint in perpetuity. It is made available under aCC-BY 4.0 International license.

Figure S1: (A) Flow cytometry data from the cell fusion experiment discussed in Figure 1D. Signal from the PE laser (RFP) is on the x-axis, and signal from the FITC laser (GFP) is on the y-axis. The first plot shows data for the genome-edited Clta-RFP parent cell line overexpressing DNM2-GFP, and the second plot shows data for the genome-edited DNM2-GFP parent cell line overexpressing Clta-RFP. Quadrants are gated to measure the enrichment of a new GFP+ or RFP+ population highlighted in the red boxes in the plot as a result of overexpression. (B) Quantification of the standard deviation from Figure1D of fluorescence intensity for both GFP-FITC and RFP-PE across 3 replicates is shown in the bar graphs. (C) Histogram of Hoechst-DAPI signal for pooled Clta-RFP and DNM2-GFP parental cell lines and the RFP+ GFP+ population from the fused condition. Quantification of the Hoechst+ population (cells with Hoechst signal beyond the peak of the pooled cells) in the pooled and the RFP+ GFP+ fused population conditions across 3 replicates is shown in the bar graph. (D) Cohort plots of successively genome-edited, dual-colored cells, three examples of clonally expanded dual-colored cells after fusion to show the intensity of clathrin (magenta) and dynamin2 (green) over time during CME events in 3 replicates.

bioRxiv preprint doi: https://doi.org/10.1101/2025.06.25.661608; this version posted June 27, 2025. The copyright holder for this preprint (which was not certified by peer review) is the author/funder, who has granted bioRxiv a license to display the preprint in perpetuity. It is made available under aCC-BY 4.0 International license.

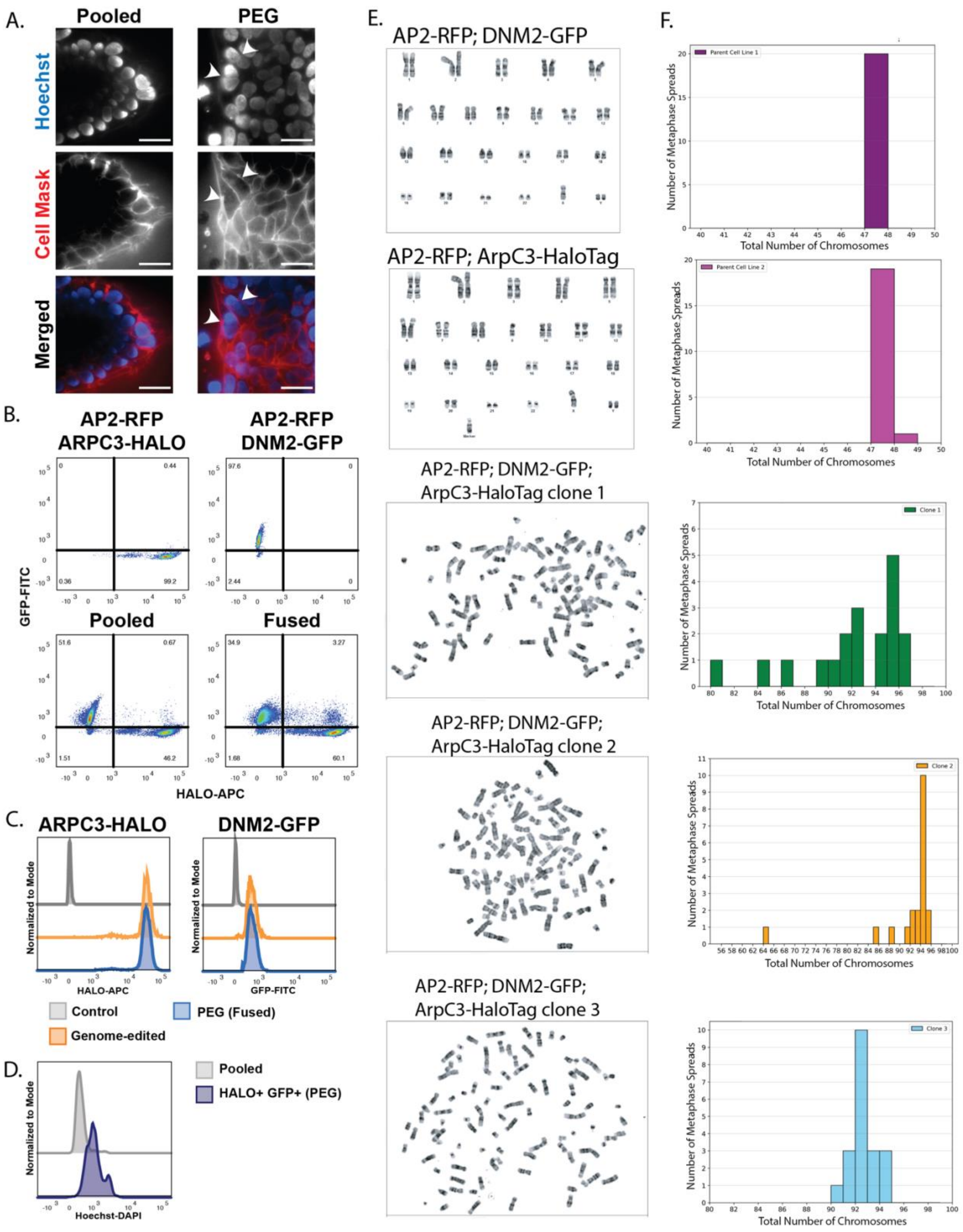

bioRxiv preprint doi: https://doi.org/10.1101/2025.06.25.661608; this version posted June 27, 2025. The copyright holder for this preprint (which was not certified by peer review) is the author/funder, who has granted bioRxiv a license to display the preprint in perpetuity. It is made available under aCC-BY 4.0 International license.

Figure S2: (A) TIRF still images on hiPSCs showing AP2-RFP; DNM2-GFP dual colored cell line co-cultured with AP2-RFP; ArpC3-HaloTag dual colored cell line without fusogen where the nuclei (blue) are clearly separated by the cell membrane (red), and the cell lines co-cultured with fusogen where some cells have multiple nuclei within one cell membrane marked by white arrowheads. Scale bar = 30 µm. (B) Flow cytometry data from the sorting of fused hiPSCs. The signal from the APC laser (HALO) is on the x-axis, and the signal from the FITC laser (GFP) is on the y-axis. The first plot shows data from the AP2-RFP; ArpC3-HaloTag parent cell line, the second plot shows data from the AP2-RFP; Dnm2-GFP parent cell line, the third plot shows data from the two parent cells co-cultured without fusogen, and the fourth plot shows data from the two parent cells co-cultured with fusogen. Quadrants are gated to measure the enrichment of a new APC+ GFP+ population in the plot, indicating co-culture and fusogen, and were used as a gate for sorting. (C) Histograms representing ArpC3-Halo and Dnm2-GFP signal for cells lacking HALO/GFP, genome-edited cells with the HALO/GFP to have a positive signal, and PEG treated HALO+ GFP+ cells. (D) Histogram showing data for the Hoechst-DAPI signal for pooled AP2-RFP; ArpC3-HaloTag and AP2-RFP; Dnm2-GFP parental cell lines and the HALO+ GFP+ population from the fused condition. Hoechst+ gating was done as in Figure S1 and used for sorting. (E) Cytogenetic analysis was performed on twenty G-banded metaphase spreads by Karyologic Inc. It appears that both parents carry an extra Y chromosome, and the AP2-RFP; ArpC3-HaloTag cell line lost a copy of chromosome 9 (top row). These cells nevertheless demonstrated normal CME dynamics, did not spontaneously differentiate, and underwent multiple freeze/thaw cycles throughout the course of this study without noticeable adverse effects on survival or physiology. The remaining karyotypes are from three different isolated single clones after two-rounds of FACS sorting. The first round of FACS sorting generated one successful clone that survived expansion, so we completed a second round of FACS sorting to isolate more single-cell clones for expansion. (E) Corresponding histogram of chromosome count from the 20 metaphase spreads. Each bar has a bin size of one.

bioRxiv preprint doi: https://doi.org/10.1101/2025.06.25.661608; this version posted June 27, 2025. The copyright holder for this preprint (which was not certified by peer review) is the author/funder, who has granted bioRxiv a license to display the preprint in perpetuity. It is made available under aCC-BY 4.0 International license.

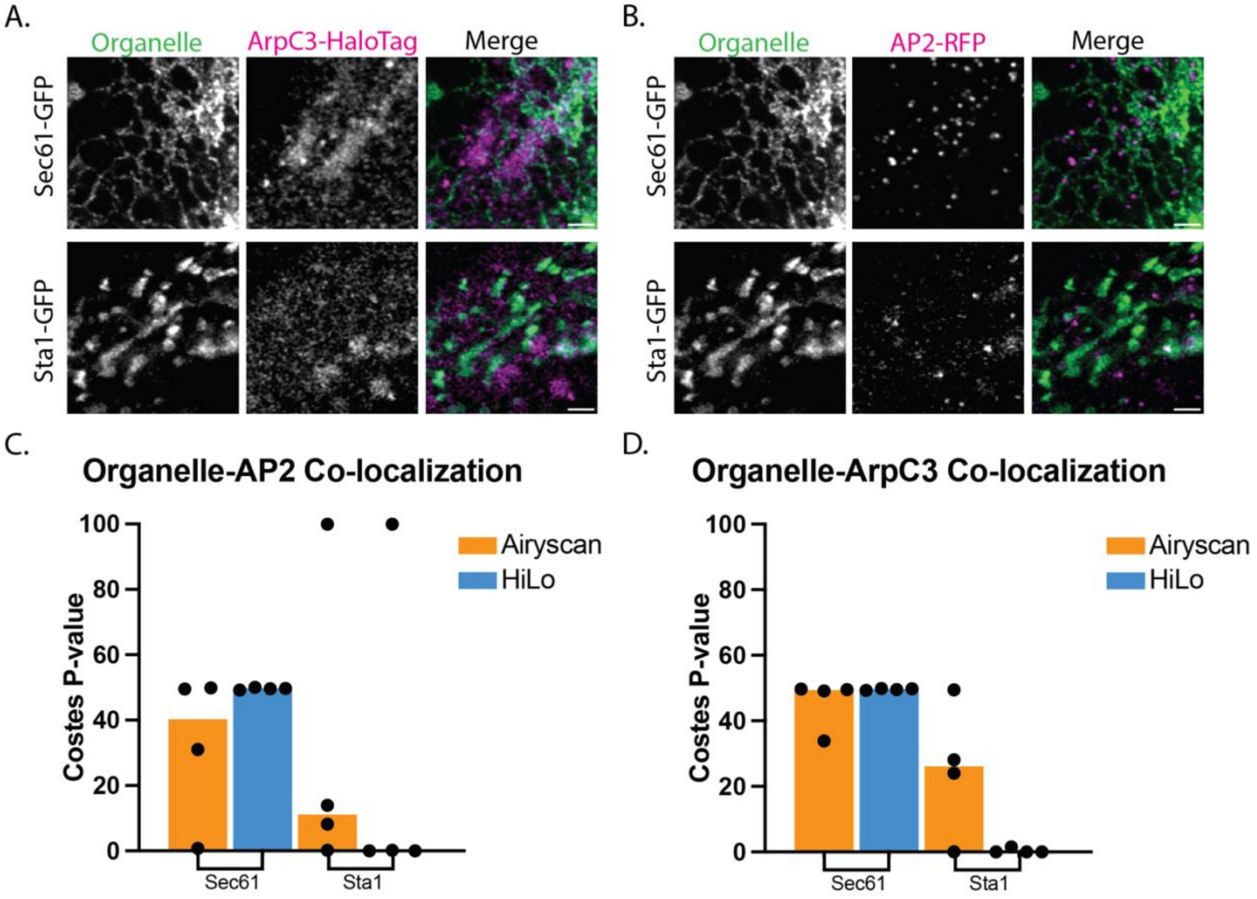

Figure S3: (A and B) Average intensity projection (z = 0.73 μm) from fixed samples of AP2-RFP; ArpC3-HaloTag cell line fused with a cell line expressing Sec61-GFP endoplasmic reticulum marker (top row) or Sta1-GFP (bottom row). Scale bar = 2 μm. (C & D) Quantification of co-localization of AP2 and ArpC3 with the Sec61 and Sta1 for both fixed and live-cell imaging using Costes randomization.

bioRxiv preprint doi: https://doi.org/10.1101/2025.06.25.661608; this version posted June 27, 2025. The copyright holder for this preprint (which was not certified by peer review) is the author/funder, who has granted bioRxiv a license to display the preprint in perpetuity. It is made available under aCC-BY 4.0 International license.

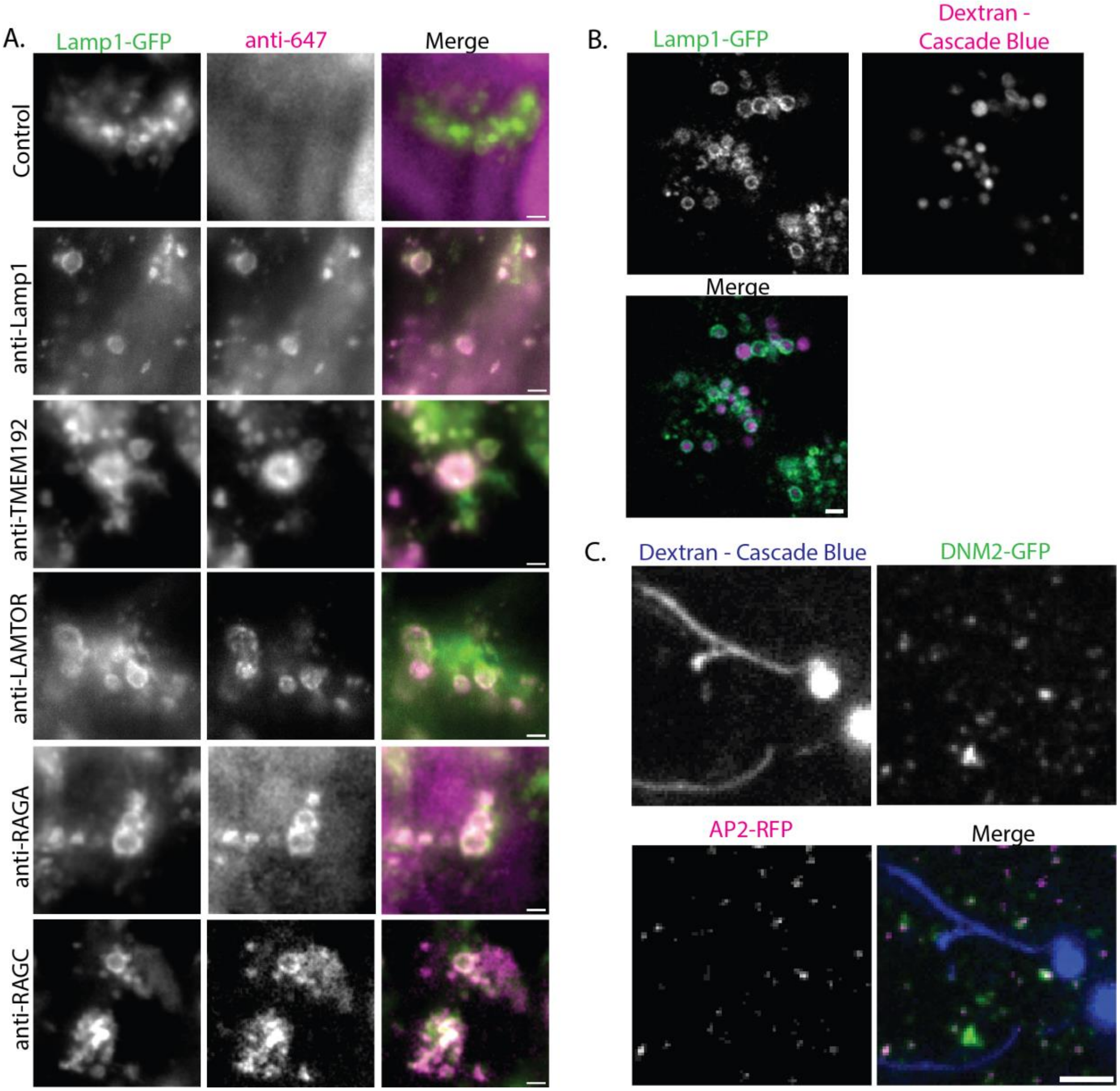

Figure S4: (A) Hi-Lo TIRF imaging of fixed hiPSC LAMP1-GFP expressing cells stained with a variety of primary antibodies to label lysosomal compartments. Scale bar = 2 µm (B) Single z-plane image of LAMP1-GFP expressing hiPSC treated with 10 kDa Dextran-Cascade blue. Scale bar = 2 µm. (C) Single z-plane image of AP2-RFP; DNM2-GFP tagged hiPSCs treated with Dextran-Cascade blue demonstrating that the Dextran can also label lysosome tubules. Scale bar = 3 µm.
